# Understanding Conformational Dynamics of Complex Lipid Mixtures Relevant to Biology

**DOI:** 10.1007/s00232-018-0050-y

**Published:** 2018-10-22

**Authors:** Ran Friedman, Syma Khalid, Camilo Aponte-Santamaría, Elena Arutyunova, Marlon Becker, Kevin J. Boyd, Mikkel Christensen, João T. S. Coimbra, Simona Concilio, Csaba Daday, Floris J. van Eerden, Pedro A. Fernandes, Frauke Gräter, Davit Hakobyan, Andreas Heuer, Konstantina Karathanou, Fabian Keller, M. Joanne Lemieux, Siewert J. Marrink, Eric R. May, Antara Mazumdar, Richard Naftalin, Mónica Pickholz, Stefano Piotto, Peter Pohl, Peter Quinn, Maria J. Ramos, Birgit Schiøtt, Durba Sengupta, Lucia Sessa, Stefano Vanni, Talia Zeppelin, Valeria Zoni, Ana-Nicoleta Bondar, Carmen Domene

**Affiliations:** 1 Department of Chemistry and Biomedical Sciences and Centre of Excellence “Biomaterials Chemistry”, Linnæus University, Kalmar, Sweden; 2 University of Southampton, Southampton, SO17 1BJ UK; 3 Max Planck Tandem Group in Computational Biophysics, University of Los Andes, Bogotá, Colombia; 4 Interdisciplinary Center for Scientific Computing (IWR), Heidelberg University, Heidelberg, Germany; 5 Department of Biochemistry, University of Alberta, Edmonton, Canada; 6 IPC, University of Münster, Münster, Germany; 7 Department of Molecular and Cell Biology, University of Connecticut, Storrs, CT USA; 8 Department of Chemistry, Aarhus University, Aarhus, Denmark; 9 Interdisciplinary Nanoscience center (iNANO), Aarhus University, Aarhus, Denmark; 10 Sino-Danish Center for Education and Research, Beijing, China; 11 UCIBIO, REQUIMTE, Departamento de Química e Bioquímica, Faculdade de Ciências, Universidade do Porto, Porto, Portugal; 12 Department of Industrial Engineering, University of Salerno, Fisciano, SA Italy; 13 Heidelberg Institute for Theoretical Studies, Heidelberg, Germany; 14 GBB Institute, University of Groningen, Groningen, The Netherlands; 15 Department of Physics, Theoretical Molecular Biophysics Group, Freie Universität Berlin, Arnimallee 14, 14195 Berlin, Germany; 16 Physiology and Vascular Biology Departments, King’s College London School of Medicine, London, UK; 17 Departamento de Física, Facultad de Ciencias Exactas y Naturales, CONICET-Universidad de Buenos Aires, IFIBA, Buenos Aires, Argentina; 18 Department of Pharmacy, University of Salerno, Fisciano, SA Italy; 19 Institute of Biophysics, Johannes Kepler University, Linz, Austria; 20 Biochemistry Department, King’s College London, London, UK; 21 Physical Chemistry Division, National Chemical Laboratory, Pune, India; 22 Department of Biology, University of Fribourg, Fribourg, Switzerland; 23 Department of Chemistry, University of Bath, Claverton Down Bath, BA2 7AY UK; 24 Chemistry Research Laboratory, University of Oxford, Mansfield Road, Oxford, OX1 3TA UK

**Keywords:** Molecular dynamics, Computational biophysics, Cell membrane, Lipid–protein interactions

## Abstract

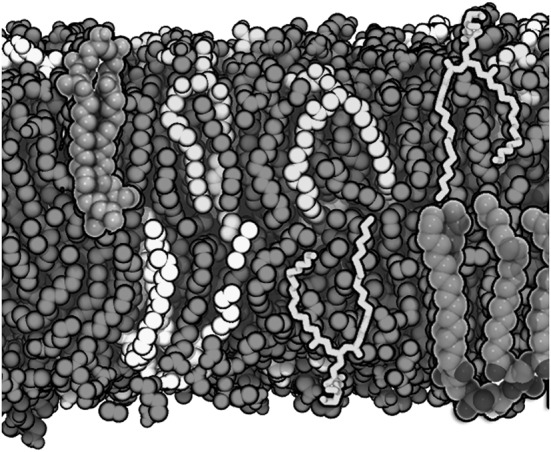

## Introduction

Membranes are ubiquitous in biology; they surround all cells and also define subcellular compartments. Consequently, they have been the focus of intense computational scrutiny for over 40 years. Lipid membranes are notoriously difficult to study using experimental methods. Progress was made in this aspect recently through high throughput structural biology and the advances in cryo-EM (Chiu and Downing 2017; Nogales 2016) and biophysical methods, particularly single molecule techniques (Ritchie et al. 2013). Computational investigations have been particularly useful for studying these systems. Studies involving computational methods are used not only to rationalise experimental data, but importantly also to predict biophysical phenomena and guide the design of new experiments. In recent years, it has become apparent, from experimental and computational data, that the lipid composition of biological membranes is a crucial aspect of their correct functioning. Computational studies have enabled the scrutiny of an enormous range of membranes, including plant (van Eerden et al. 2017a), eukaryotic (Ingólfsson et al. 2014), bacterial (Hsu et al. 2017), and archaeal membranes (Pineda De Castro et al. 2016b).

The computational techniques that have been employed for these studies include molecular dynamics (MD), Monte Carlo (MC), dissipative particle dynamics (DPD) and a plethora of methodologies for enhanced sampling and calculation of free energies. In addition to these simulation methods, other physics-based theoretical methods and bioinformatics techniques have also been used. Atomistic and united atom models have provided some remarkable insights into protein–ligand, protein–protein, and protein–lipid interactions as well as the conformational dynamics of the individual macromolecules that comprise biological membrane systems. Given the generally substantially larger size of membrane systems compared to systems composed of just proteins in solution, coarse-grain (CG) models are almost as popular as atomistic/united atom models for simulations of membranes (Orsi and Essex 2011; Marrink et al. 2007; Chu et al. 2006). An added advantage of CG methods, in addition to fewer interaction centres and thus fewer calculations, is the faster kinetics that are observed due to the smoother energy landscapes which arise from the reduced system dimensionality. Increasingly, both resolutions are employed to study the same molecular system, thereby combining the advantages of both and overcoming their respective limitations. For example, CG simulations were used to identify the preferred membrane location of a transmembrane or peripheral membrane protein and the protein–membrane interactions, whereas the conformational dynamics of the protein and interactions with other molecules were studied using fine-grained resolution (Stansfeld and Sansom 2011; Piñeiro et al. 2011). Hybrid multiscale simulations in which portions of the system are represented at CG resolution while other parts are described at a fine-grain resolution have also been reported (Genheden and Essex 2015; Kar and Feig 2017; Kuhn et al. 2015). Bioinformatics methods such as prediction of transmembrane regions of membrane proteins and phylogenetic studies provide an important complement to simulation studies, in particular when structures are either incomplete or not available at all (Tian et al. 2018; Wang et al. 2018). With the advent of high throughout sequencing methodologies, such data will be available in large amounts and therefore it can be expected that such approaches, which are often based on data-mining, will play an increasingly significant role.

In the following article, we review recent progress in our understanding of the implications of the compositional heterogeneity of biological membranes that were presented in a Centre Européen de Calcul Atomique et Moléculaire (CECAM) meeting that took place in Lugano (Switzerland) in January 2018.

## Simplified Models of Lipid Membranes and Their Components

Simplified models of membranes are useful when there is a need to study systems that are too big or too complex to be modelled atom-by-atom, or where there is a need to simulate systems for periods that are longer than a few microseconds (although specialised hardware can be use to overcome this limit (Shaw et al. 2008)). Simplified models range from continuum approaches to lattice models, followed by phenomenological coarse-grained models (Friedman et al. 2009) and finally traditional coarse-grain models such as the very popular MARTINI force field (Marrink et al. 2007). Nowadays, applications range from the traditional (membranes and membrane proteins) to complex and very heterogeneous membrane structures and membrane particles. Several examples are given here.

The state of the art in simplified models of lipids is exemplified here by four contributions, "Domain Formation in Lipid Membranes, Studied by Lattice Models", "Modelling the Complexity of Thylakoid Membranes", "Towards a Molecular View of Lipid Droplet Biology", and "Coarse-Grain Approaches to Study Block-Copolymer Nanoparticles for Drug Delivery Systems". In the first of these, a lattice model is reviewed, which can yield tremendeous advantage in speed. The other three report on studies where CG and MD were used to deal with complex and very different systems: the thylakoid membrane, lipid droplets and block-copolymer nanoparticles. These studies demonstrated how such simulations can be used for investigations that involve heterogeneous membranes in biology, complex membrane structures, and biotechnological applications.

### Domain Formation in Lipid Membranes, Studied by Lattice Models

Domain formation in lipid membranes is of major relevance for signal-processing in cells. The lipid-raft theory (Lingwood et al. 2009) postulates the presence of liquid-ordered ($L_{{\text{o}}}$) and liquid-disordered ($L_{{\text{d}}}$) domains. Attempts to analyse the formation of these domains via MD simulations were initially carried out based on coarse-grained modelling (Risselada and Marrink 2008; Hakobyan and Heuer 2014) with the MARTINI force field (Marrink et al. 2007). Later on, it could be shown that with time-consuming MD simulations the onset of the domain formation could also be obtained from united atom modelling, which required simulation times of more than 10 μs (Hakobyan and Heuer 2013). A recently introduced 2D-lattice-based approach was used to exceed the previous limitations in timescale and length scale by orders of magnitude (Hakobyan and Heuer 2017). At the same time, it allowed the extraction of the underlying information about the thermodynamic origin of domain formation.

In the 2D-lattice-based approach, a model is devised where each point on a lattice corresponded to one lipid. The lattice can represent either pure lipid membranes or lipid mixtures. So far, pure DPPC and DLiPC membranes as well as DPPC/DLiPC mixtures have been systematically explored (Hakobyan and Heuer 2017). Several key aspects of model design can be addressed based on an analysis of MD simulations. The first of these is the choice of an appropriate number of nearest neighbours, which defines whether the lattice shall be hexagonal or quadratic. The radial distribution function of the head groups of the lipids, calculated from MD simulations, revealed that each lipid had four nearest neighbours, which suggested the use of a quadratic lattice. The second issue that needs to be addressed is how to characterise the individual lipids in terms of their degrees of freedom. A useful property in this respect is the deuterium-order parameter of the carbon tails, calculated from MD simulations. For example, the order parameters characterise the difference between the gel phase and the disordered phase for pure DPPC. Finally, the free energy of the system can be quantified by three major contributions that can be extracted from an initial MD simulation. These are the potential energy between adjacent lipids, the potential energy of individual lipids (mainly the interaction of both chains), and the configurational entropy of the chains.

**Fig. 1:**
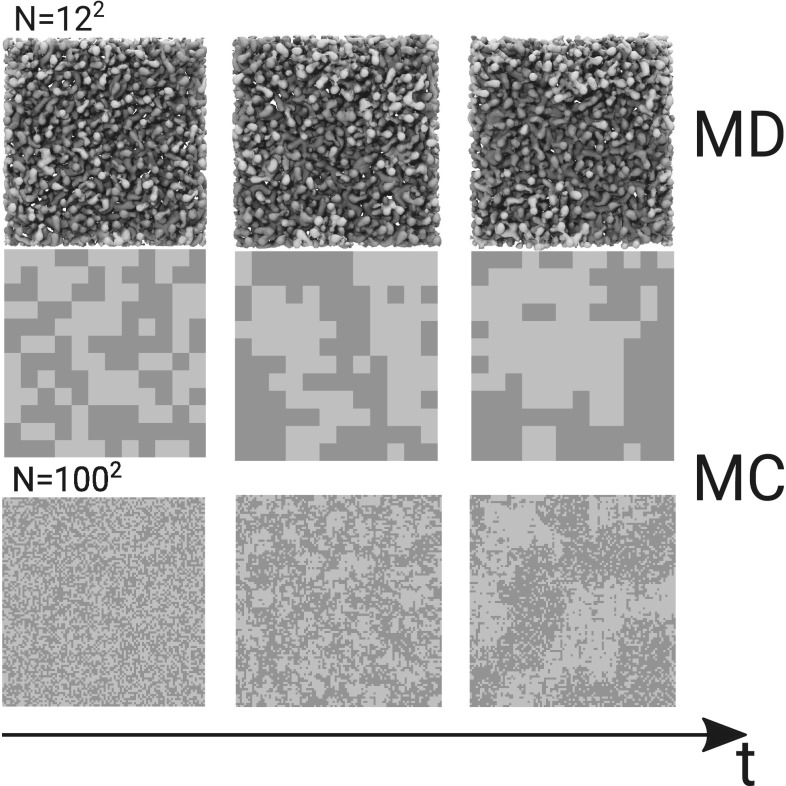
Membrane simulations with a lattice model. Time evolution of a DPPC(pink)/DLiPC(cyan)-mixture at $T= 290$ K, based on all-atom MD simulations (with a system size of $12^2$) and MC simulations of the corresponding lattice model (with a system sizes of $12^2$ and $100^2$). The simulations were started with random distributions of both lipid types. A comparison of the MD and MC simulations revealed a qualitative agreement with respect to the unmixing process (upper and middle frames). Such an agreement was even found on a quantitative level (Hakobyan and Heuer 2017). Importantly, the MC simulations were eight orders of magnitude faster than the MD simulations. Variation of the system size of the lattice model (lower frame) betrayed the impact of finite size effects on the domain formation. Interestingly, for simple DPPC/DLiPC mixtures, it turned out that the initial process of unmixing does not display relevant finite size effects for system sizes larger than $12^2$

The equilibrium properties of the lipids in the lattice model, as well as their approach towards equilibrium, are studied via MC simulations. Whereas the potential energy of the model can be calculated directly based on MD simulations of a corresponding system, the configurational entropy of the chains as a function of the order parameter is not known. To this end, an initial MC simulation is started with a generic choice of the entropy function and the resulting Boltzmann distribution of order parameters is compared with the observed distribution. The deviation between the two suggests how the entropy function should be modified. After several iterations, with subsequent updates of the entropy function, the MC- and MD-order parameter distributions should become almost identical, indicating the convergence of the entropy function. Once the complete information for the lattice model is available, a more detailed comparison between MD and MC is possible (Fig. 1). The lattice model has two advantages over MD: it can be used to reach timescales which are many orders of magnitude longer and to study systems that are much larger in size. Such lattice-based MC simulations can therefore be used, e.g., to follow on the dependence of the domain formation on the chosen system size and to analyse the possibility of critical behaviour close to the transition between different membrane phases.

The grid-based approach can be expanded to incorporate membrane proteins, stochastic interaction effects with the surrounding medium, and curvature effects, just to give a few examples. Of particular interest is the addition of cholesterol which would enable the study of lipid rafts. Work along this line is in progress.

### Modelling the Complexity of Thylakoid Membranes

The thylakoid membrane, found inside chloroplasts and in the cytosol of cyanobacteria, is essential for most forms of life. It has the special capability to perform photosynthesis, the process in which solar energy is harvested and converted into biochemical energy. The protein complex photosystem II (PSII), embedded in the thylakoid membrane, is a key component in this process. PSII uses chlorophylls and carotenoids as antennas to capture photons. The energy of the photons is used to oxidise water and to subsequently reduce plastoquinone (PLQ) to plastoquinol (PLQol), which renders oxygen as a waste product. Concurrently a proton gradient is established, which is used for the generation of adenosine triphosphate (ATP). PSII functions as a homodimer, where each monomer consists of 27 subunits in plants and 20 in cyanobacteria, respectively. A large number of cofactors supplement PSII with its light-harvesting and water-splitting capabilities. Each monomer contains around 77 cofactors, including ions and a number of glycolipids.

**Fig. 2:**
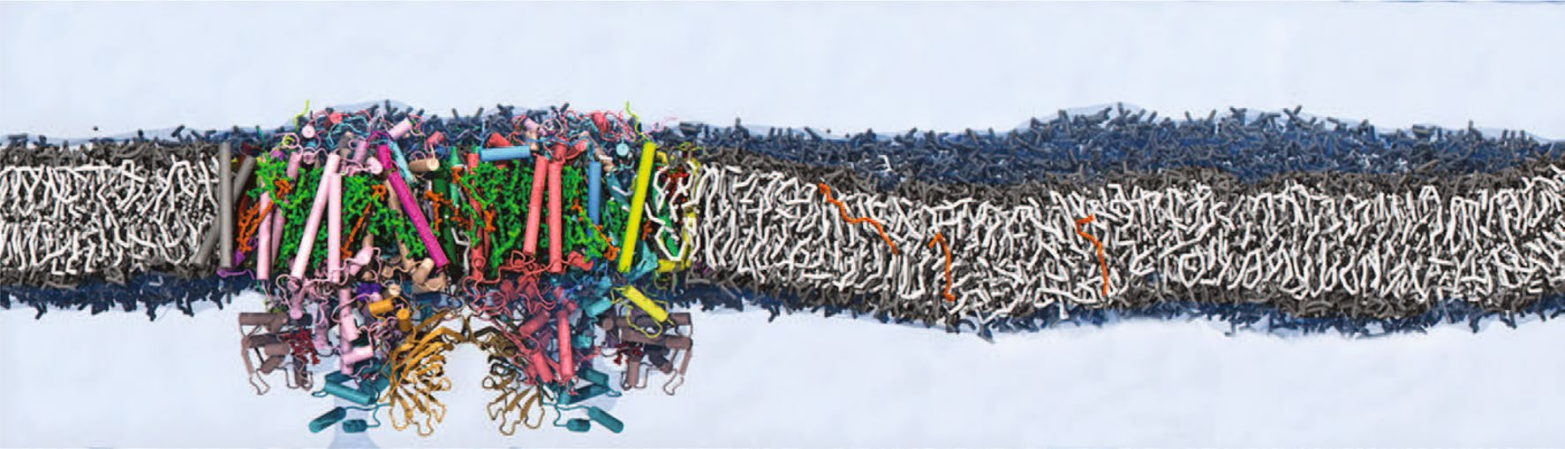
MARTINI model of PSII embedded in the thylakoid membrane. The PSII dimer is shown with different colours for its subunits. Chlorophylls are depicted in green, *β*-carotenes in dark orange, haems in red and the exchanging PLQ electron carriers in orange. The thylakoid lipids are shown with white tails and grey heads. Water is rendered as blue translucent surface

To provide a dynamical view on this large and important protein complex, a CG model of PSII from cyanobacterium *Thermosynechococcus vulcanus* was developed (van Eerden et al. 2017a) based on the MARTINI force field, Fig. 2. The complex was embedded in a realistic thylakoid membrane composed of a mixture of phosphatidylglycerol (PG), and the glycolipids digalactosyldiacylglycerol (DGDG), monogalactosyldiacylglycerol (MGDG) and sulfoquinovosyl diacylglycerol (SQDG). Validation of the lipid parameters was performed against reference atomistic simulations. At both levels of resolution, the different thylakoid lipid types were well mixed in the plane of the membrane under conditions where the lamellar state was stable (van Eerden et al. 2015). Similarly, models for the cofactors were optimised with respect to atomistic models (de Jong et al. 2015).

Simulations of 60 μS of the full PSII dimer in the thylakoid membrane revealed a robust complex with a stable ternary and quaternary structure (van Eerden et al. 2017a). Overall, the protein subunits and cofactors were more flexible towards the periphery of the complex and near the PLQ exchange cavity and at the dimer interface. Of all cofactors, *β*-carotenes showed the highest mobility. Some of the *β*-carotenes diffused in and out of the protein complex via the thylakoid membrane, which suggested the existence of a dynamic exchange pool of these cofactors that are crucial for protection of PSII against photobleaching.

A cofactor that is most definitely mobile is PLQ, which acts as an electron carrier between PSII and the cytochrome b6f complex. To understand how PLQ enters and leaves PSII, a series of CG MD simulations covering a total simulation time of more than 0.5 ms was performed (van Eerden et al. 2017b). The long timescale allowed the observation of many spontaneous entries of PLQ into PSII, as well as the unbinding of the reduced carrier PLQol from the complex. The data confirmed the hypothesised existence of two exchange channels connecting the bulk thylakoid membrane to the PLQ exchange cavity. Surprisingly, a third hitherto unknown exchange channel was discovered in the simulations. The results revealed that a promiscuous diffusion mechanism exists, in which all three channels function as entry and exit channels. The exchange cavity itself serves as a PLQ reservoir. The thylakoid lipids also play a dynamic role in this process, as they can leave and enter the exchange cavity. This enables the exchange of co-crystallised lipids with the bulk membrane and suggests a more open nature of the PLQ exchange cavity. Interestingly, an accumulation of MGDG and SQDG lipids in the annular shell around the protein, forming distinct binding sites, was also observed in MD simulations (van Eerden et al. 2017c). The biological relevance of this finding, however, remains unclear.

Taken together, the simulations of PSII discussed above provide a direct view of the dynamic organisation inside and around the PSII complex, in particular the exchange of electron carriers, a key step of the photosynthesis machinery. The CG model of PSII paves the way for future studies aimed at unravelling the large-scale organisation of thylakoid membranes including the light-harvesting antenna complexes, and eventually, the full chloroplast organelle. In addition, simulations with full atomistic details can shed light on events that take place on shorter timescales and require information about the transient assembly of protein/water hydrogen bond networks (Guerra et al. 2018).

### Towards a Molecular View of Lipid Droplet Biology

Lipid droplets are intracellular organelles that are important for energy storage, generally in the form of neutral lipids such as triacylglycerols (TG) or steryl esters (SE). These neutral lipids behave essentially as pure oil, and lipid droplets can be thus considered as intracellular emulsions. Their oily core is surrounded by a monolayer of phospholipids (PLs) that act as surfactants in the aqueous environment of the cytosol.

Despite this unique structure, numerous proteins that are known to localise at the surface of lipid droplets can also be found in other organelles, most notably the endoplasmic reticulum (ER) and the Golgi apparatus. Thus, it is important to understand the differences and similarities between the surface of lipid droplets and that of lipid bilayers, in order to understand how proteins are able to bind membranes with such different molecular structures and how protein trafficking might be regulated inside the cell (Vanni 2017).

**Fig. 3:**
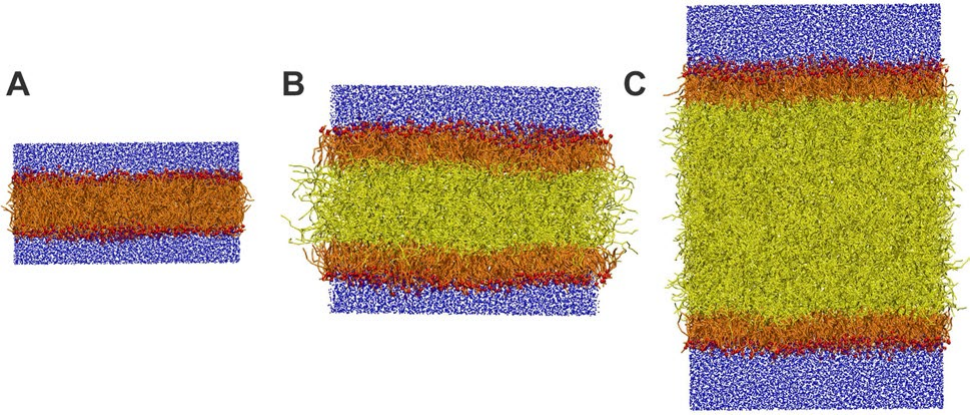
Simulations of lipid droplets with a coarse-grained model. Screenshots from MD simulations of a lipid bilayer (**a**) and of trilayers mimicking the lipid droplet structure with different amounts of oil (**b**, **c**) are shown as discussed in (Bacle et al. 2017). Water is coloured in blue, PLs in orange and TG in yellow. The beads that correspond to the phosphate group are highlighted in red. The CG lipid model by Klein and coworkers was used, and all the simulations were performed using the software LAMMPS (Plimpton 1995). All the systems are constituted by 800 POPC molecules with 816 TG molecules in system (**b**) and 2448 TG in system (**c**). System (**a**) and (**b**) contain 15400 water molecules and system (**c**) 27000. All the simulations were run for 500 ns

Lipid droplets have sizes of 100 nm–100 μm and can thus be considered flat on a molecular scale. Hence, one of the typical model systems to study their surface in MD simulations is a trilayer, where a thick layer of neutral lipids is sandwiched between two monolayers of PLs (Fig. 3). MD simulations were used to compare trilayers having different sizes and different values of surface tension (ST) to bilayers. Furthermore, models with different resolutions were used: united atom (UA) and CG. TG parameters consistent with the Shinoda-De Vane-Klein CG force field were developed for simulations with a CG model, since this force field was explicitly developed to correctly reproduce the surface and interfacial tensions of the surfactants and PLs (Shinoda et al. 2010).

One of the main results of the simulations was that at low values of ST, the presence of oil did not have a major effect on classical surface properties, including area per lipid, monolayer thickness or order parameters (Bacle et al. 2017). Of note, whereas the bilayer ST is usually very low, this is not the case for emulsions. A recent study showed that interfacial tension of purified lipid droplets is around 2–4 mN/m, i.e., 1–2 orders of magnitude higher than that of bilayers (Ben M’barek et al. 2017). Thus, whether ST can play a role in modulating surface properties of lipid droplets was also investigated. This was performed by focusing on two properties, interdigitation and lipid packing defects. Interdigitation is a measure of chain overlap between two layered lipid phases, which in this case were monolayer PLs and the core TGs. Lipid packing defects are the interfacial voids at the membrane–water interface where the hydrophobic chains of PLs are transiently and unfavourably in contact with water. An interesting bimodal behaviour was revealed when packing defects and interdigitation were examined: a slow increase in lipid packing defects and interdigitation was shown below ST $\approx$ 10 mN/m, whereas a sharp increase was observed above this threshold (Bacle et al. 2017).

To further investigate the role of ST, CG simulations were combined with in vitro and cellular approaches to study the mechanism of lipid droplet biogenesis, and in particular the budding of lipid droplets from the ER, where they initially form. Decreasing the ST was found to promote the budding of lipid droplets via the spontaneous dewetting of oil from the bilayer. Lipid composition of the ER played a critical role in influencing this process (Ben M’barek et al. 2017).

Taken together, simulations of lipid droplets showed that ST plays an important role in lipid droplet formation and protein targeting to them. On a more technical note, while the increase in computational power and the development of new modelling approaches give us the possibility to simulate large systems and complex phenomena (Soares et al. 2017), an accurate description of interfacial properties of liquid–liquid interfaces remains challenging. Nevertheless, by choosing the appropriate parameters and conditions, MD-based approaches can be successfully used to study such complex lipid mixtures in relevant biological contexts.

### Coarse-Grain Approaches to Study Block-Copolymer Nanoparticles for Drug Delivery Systems

The development of drug delivery systems (DDS) for specific applications has proven to be quite a challenge. Knowledge of the mechanism of drug encapsulation and release at the atomic or molecular level can help in the design of efficient encapsulation systems, according to the desired objectives for each particular case. Furthermore, many drugs need to cross a biological barrier in order to access their site of action. Design of DDS thus requires a solid understanding of the drug/lipid bilayer interactions. Computer simulations can be very useful in this respect. Relevant information on the system can be obtained using MD simulations at different length- and timescales, from the continuous to the atomistic level.

During the past decades, a wide range of drug delivery systems has been developed, including liposomes, lipid solid nanoparticles and metallic nanoparticles. Systems based on biocompatible amphiphilic block-copolymers are interesting in this respect. Such systems are comprised of tunable materials that, depending on their block length and the hydrophilic/hydrophobic balance, can assemble into different structures. MD simulations were hence used to study nanoparticles based on amphiphilic copolymers for different DDS applications (Grillo et al. 2017).

Poloxamers/pluronics (PL) are linear non-ionic triblock (ABA-type) copolymers. The *B* block is composed of hydrophobic poly(propylene oxide), PPO. The two *A* blocks are hydrophilic poly(ethylene oxide), PEO, homopolymers. By changing the length of the polymer blocks, their solubility and other thermodynamic properties can be customised for specific applications. Furthermore, due to the PEO composition, the DDS can be made non-immunogenic and thus biocompatible. Interestingly, some experimental studies have shown that poloxamers sensitise multi-drug resistance cells, increasing the cytotoxic activity of anti-neoplasmic drugs. This could be due to inhibitory effects on P-glycoprotein pumps that may indirectly be attributed to interactions with the lipid membranes.

**Fig. 4:**
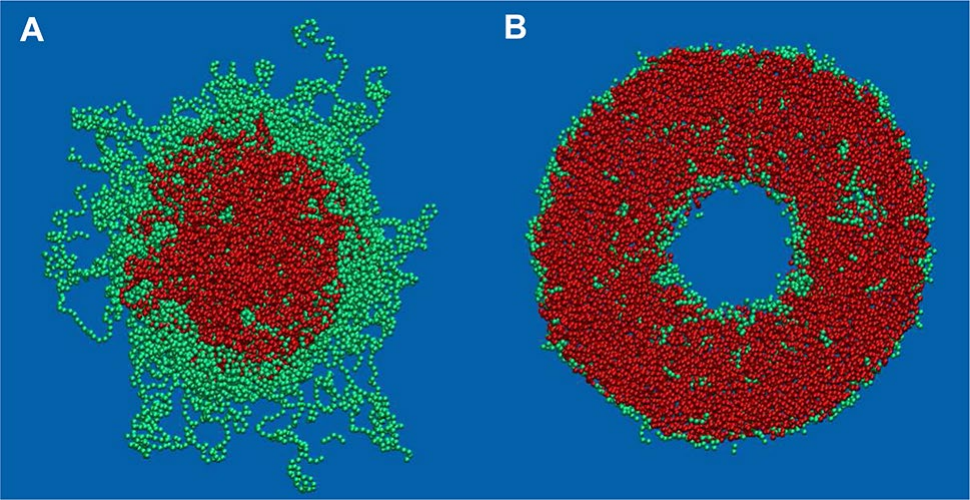
Block-copolymer nanoparticles. Representative snapshot for the simulated: **a** F127 micelle and **b** L121 polymersome. PPO and PEO bead are coloured red and green, respectively. PW waters are coloured blue. CG simulations of up to 1.5 μs were performed using GROMACS 4.5 (Berendsen et al. 1995; van der Spoel et al. 2005) as detailed in (Wood et al. 2018)

Two poloxamer nanoparticles: micelles (PL-F127) and polymersomes (PL-L121) were studied by CG MD simulations. In simulations of PL-F127, one hundred PL-F127 molecules were arranged in a pre-assembled plain micelle (Fig. 4a), with their hydrophobic PPO beads (red) inside the core surrounded by the PEO ones (green). The micelle was solvated in polarisable MARTINI PW water. The overall organisation of the nanoparticle showed that the PPO blocks form a hydrophobic core, with no contact with the aqueous face. Around the PPO core, PEO blocks adopted an extended distribution, with little access to the hydrophobic region. In addition, the PEO blocks formed a PEO–water interface (Wood et al. 2018).

To examine PL-F127 micelles as potential nanocarriers, an anti-migraine drug (sumatriptan) was encapsulated in such micelles. Sumatriptan is a serotonin receptor agonist that is active in the brain and needs to cross the blood–brain-barrier. Encapsulating it in s DDS could be a way to improve its clinical efficacy. The dynamics of the sumatriptan encapsulation was therefore followed through different simulations. The parametrisation of the sumatriptan CG model was validated with atomistic simulations and small angle X-ray scattering (SAXS) experiments (Wood et al. 2018). The simulations accounted for the drug’s interfacial preferential distribution. Moreover, analysis of the simulations yielded molecular insights on dynamic light scattering (DLS) and Z-potential experiments (Wood et al. 2018). Preference of sumatriptan to interfaces had also been observed in atomistic simulations of sumatriptan in lipid bilayers. There, specific interactions with the lipid bilayers were identified including cation-$\pi$, salt bridges and hydrogen bonds that anchored the drug to the lipid membrane and limited its diffusion through the membrane (Wood and Pickholz 2013).

Polymersomes are similar to liposomes due to their core-shell structure and can encapsulate both hydrophilic and hydrophobic molecules (Grillo et al. 2017). A polymersome was built up with 936 PL-L121 molecules, arranged in vesicle shape as illustrated in Fig. 4b. The plain polymersome was stable for at least 1.5 μs. The potential of the polymersomes to encapsulate oncological drugs was explored using the poorly soluble anticancer drug paclitaxel (Namgung et al. 2014). In the simulations, paclitaxel had initially been placed in the water phase. It penetrated the hydrophobic core of the polymersome (within $\sim$ 300 ns). Inside the polymersome, the drug molecules formed small aggregates.

Overall, MD simulations are useful in studies of DDS nanoparticles. On top of the examples listed above, another such system is high-density lipoprotein particles (nanodisks) used to deliver hydrophobic drugs to impaired cells and tissues. Moreover, synthetic bilayers of block-copolymers could be used to study membrane proteins. Here, the bilayer permeability could be tuned in order to warranty the passage of ions through an ion channel.

## The Effects of Complex Curvature Environments in Heterogeneous Lipid Mixtures

Cellular membranes adopt a range of curved morphologies and undergo dynamic shape transitions during cellular processes such as cell division, endocytosis and exocytosis. The lipid compositions of different cellular membranes vary widely. Understanding how different lipid species and their local concentrations modulate equilibrium configurations and tendencies to undergo curvature changes remains an open and challenging question. Mitochondria are critical energy generating organelles in eukaryotic cells whose proper functions depend on maintaining complex curved membrane morphologies. The mitochondrion is characterised by a double membrane structure, where the inner membrane consists of a flat inner boundary region, highly curved invaginations (cristae), and connecting tubular structures called cristae junctions. Disruption of the cristae shape and size is observed in several mitochondrial metabolic diseases.

**Fig. 5:**
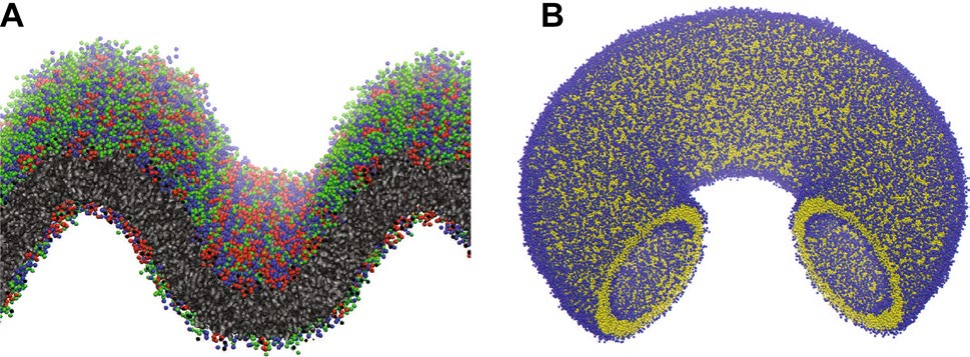
Curved bilayer structures. **a** A buckled bilayer containing different lipid species. Buckled bilayers were simulated in GROMACS 5 with the MARTINI 2.2 Forcefield at 303 K. Buckled bilayers were generated by applying lateral pressure along the long dimension of the bilayer. From the buckling trajectories snapshot at fixed lateral strains was extracted and run with constant number of particles, volume and temperature (*NVT*) for 36 μs to allow for lipid species partitioning in curvature space. **b** A ring torus with a wedge removed to show the inner surface. This shape was generated using the tool BUMPy from a flat MARTINI model bilayer

A key component in mitochondrial function is the unusual lipid cardiolipin. Cardiolipin is a tetra-acyl phospholipid. In eukaryotes, it is primarily found in the mitochondrial inner membrane, making up approximately 20% of the total inner membrane lipid composition. Cardiolipin is known to play a role in the maintenance of the highly curved state of the inner membrane as well as spatial organisation of the mitochondrial protein complexes required for respiration and oxidative phosphorylation. The spatial organisation of cardiolipin in membranes of varying curvature was investigated using MD simulations with the coarse-grained MARTINI force field (Marrink et al. 2007). Buckled bilayers (Fig. 5a) were generated through the application of lateral pressures. This yielded curved membrane structures which resemble mitochondrial cristae, though at smaller length scales and at higher curvatures. Through the induction of buckled states and investigations of curvature concentration coupling in buckled states, it was possible to evaluate how the presence of cardiolipin affected the mechanical properties and lipid dynamics in multicomponent (binary and ternary) lipid bilayers (Boyd et al. 2017).

The results of these simulations showed that incorporation of cardiolipin increases bilayer deformability and that cardiolipin becomes highly enriched in regions of high negative curvature. It was also shown that another mitochondrial lipid phosphatidylethanolamine (PE), which is known to have a negative spontaneous curvature, does not partition as strongly to regions of negative curvature, and does not increase the deformability of the membrane in a significant manner. These differences between the properties or cardiolipin and PE could not be inferred based upon molecular geometry considerations, and therefore, it is likely that a more sophisticated theory and further analysis will be required to understand the underpinning of this observation.

Cardiolipin is generated in the mitochondria through a multi-step synthesis that involves several enzymes and intermediates. A final step in the production of mature cardiolipin is a transacylation reaction where saturated acyl chains are replaced by unsaturated chains. The tafazzin enzyme is involved in this process, and mutations to the tafazzin encoding gene can lead to several clinical disorders which affect the heart and/or skeletal muscle tissue. A phenotype of this class of disorders can be the presence of abnormal mitochondrial structures including a lack of well-defined cristae structures. One of the biochemical consequences of a malfunctioning tafazzin enzyme is the accumulation of the cardiolipin intermediate species monolysocardiolipin (MLCL), which contains only three acyl chains. Again, using the MD simulations of heterogeneous buckled bilayers with the MARTINI model, as well as atomistic simulations of homogeneous flat bilayers, the link between the accumulation of MLCL and the stability of curved bilayer morphologies was investigated (Boyd et al. 2018). Atomistic MD simulations revealed differential headgroup dynamics between cardiolipin and MLCL. Moreover, increased cohesiveness of MLCLs solvent interfacial region was found, which may have implications for protein organisation. Based on CG simulations, substitution of MLCL for cardiolipin in bilayers mimicking mitochondrial composition does not increase membrane deformability in the same manner as native cardiolipin; instead, bilayers containing 20% MLCL behave similarly to binary PC/PE bilayers. Furthermore, the curvature-dependent partitioning behaviour observed for cardiolipin is not observed to the same extent for MLCL. The results of these simulation studies have revealed differences between cardiolipin and MLCL at the molecular and mesoscopic levels that may help establish a physical mechanism that gives rise to disease states associated with defects in cardiolipin remodelling.

As computational power continues to increase, our ability to simulate membrane systems which more closely match biological membranes, in terms of composition, size, and shape, should also increase. With this in mind, current scientific tools for the purpose of generating bilayers of varying shape and size and composition have several limitations, one of which being the inadequacy of properly balancing lipid ratios between leaflets in a curved bilayer. A technique for creating starting structures for MD simulations of curved membranes has recently been developed based on geometric transformations of flat bilayers. This tool, named BUMPy (Building Unique Membranes in Python), allows for generation of any number of curved shapes and is a forcefield independent method that can be applied to both CG and atomistic systems. The key to the method is based upon determining the location of the monolayer pivotal plane (Wang and Deserno 2015). This allows for both the lipid area densities and inter-leaflet lipid ratios to be correctly and precisely determined. Using this tool, it is possible to generate a variety of complex curved systems by coupling flat, spherical, tubular, and toroidal elements together. The tool will enable the investigation of biologically and theoretically fascinating shapes, such as the torus (Fig. 5b), which has a varying Gaussian curvature over its surface. BUMPy is implemented in Python as a command-line tool. It can be freely downloaded at www.github.com/MayLab-UConn/BUMPy, with usage details and examples.

## Lipid–Lipid, Lipid–Water and Lipid–Water–Solute Interactions

Lipid–lipid interactions not only shape the membrane structure and its mechanical properties but also how it is modified. Such interactions are also mediated by water (Karathanou and Bondar 2018), salts (Friedman 2018; Pineda De Castro et al. 2016a), and other solutes, including drug molecules (Karlsson et al. 2013; Coimbra et al. 2017). Simulations enable us to follow on lipid–lipid interactions in great detail and even follow on transitions between states or domains within the membrane. This makes simulation studies indispensable when studying lipid–lipid interactions. Purely theoretical studies can also be insightful in the study of lipid interactions—one such study is reported in "Registration of Lipid Domains from the Two Membrane Leaflets" section.

### Registration of Lipid Domains from the Two Membrane Leaflets

Phase separation in biological membranes plays an important role in protein targeting and transmembrane signalling. Commonly, domains with similar phases occupy matching positions in opposing monolayers. Two alternative hypotheses explain such domain registration: (i) lipid interactions at the membrane midplane and (ii) minimisation of elastic membrane deformations. The first hypothesis asserts that lipid layers interact at the membrane midplane in the same way that ordered ($L_{{\text{o}}}$) and disordered ($L_{{\text{d}}}$) phases in one leaflet interact at their interfaces (Collins 2008). It further specifies that a quantitative estimate for the elastic interaction energy $E_\text{midplane}$ can be made taking into account the line tension $\gamma$ that exists between ordered and disordered domains: $E_\text{midplane}=\gamma /h$, where *h* is the thickness of the monolayer. Assuming that $\gamma \approx 5$ pN and *h* = 2.5 nm, $E_\text{midplane}$ was estimated as $E_\text{midplane} \approx 2$ pN/nm $\approx$ 0.5 kT/nm^2^ (Collins 2008).

**Fig. 6:**
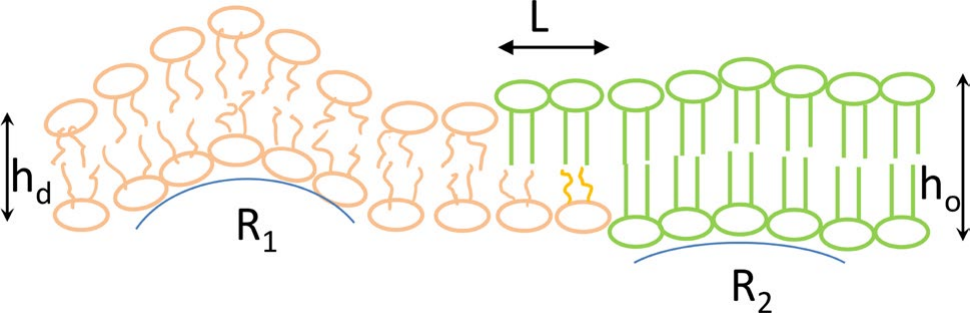
Registration of lipid domains. Ordered domains (green lipids) from the two leaflets are always in register. The alignment is driven by both (i) the line tension around ordered domains and (ii) membrane undulations. The line tension occurs as a result of hydrophobic mismatch between the lipids in the thicker (bilayer thickness $h_{{\text{o}}}$) ordered domain and the lipids in the thinner (bilayer thickness $h_{{\text{d}}}$) disordered domain (orange). It induces elastic deformations of the lipid molecules adjacent to the border of both phases that minimise the access of water to the hydrophobic acyl chains. A stepwise transition from $h_{{\text{o}}}$ to $h_{{\text{d}}}$ reduces the energetic costs for the shape changes of the lipids. It is realised by spatially separating the height changes in the two leaflets from each other, i.e., by introducing a small shift *L* between the edges of the ordered domains in the two leaflets (Galimzyanov et al. 2015). The wavelength of membrane undulations depends on the local resistance to lipid deformation. Owing to their smaller splay modulus, disordered lipids populate areas with larger monolayer curvature (*R*_1_). In contrast, stiffer ordered domains localise to areas with lower curvature (*R*_2_), which naturally coincide with the opposing monolayers (Galimzyanov et al. 2017)

This approach may seem counter-intuitive, since it ignores the molecular origin of $\gamma$. The hydrophobic effect requires that the lipids at the interface between the thicker ordered domain and the thinner disordered domain undergo elastic deformations in order to exclude water from the interface (Fig. 6). Thus, an energy penalty (in the form off line tension) associated with elastic lipid deformations arises only at the water exposed side of the $L_{{\text{o}}}$/$L_{{\text{d}}}$ interface. Since the acyl chains are well hidden from water at membrane midplane, the hydrophobic effect does not drive lipid deformations or spatial lipid rearrangements in case an $L_{{\text{o}}}$ domain in one leaflet is facing an $L_{{\text{d}}}$ domain in the other. Since an energetic penalty is not incurred, $E_\text{midplane}$ cannot drive domain registration.

Lipid interdigidation is another form of interaction at the membrane midplane. The tails from lipids in the $L_{{\text{d}}}$ phase are envisioned to cross beyond the midplane of the bilayer and interlock with the tails of the same phase in the opposing leaf. This process is accompanied by a gain in lipid chain entropy, which can be assumed to be the driving force for the phase separation (since interdigidation is not possible with the denser packed lipids of the $L_{{\text{o}}}$ phase). However, this hypothesis is in stark contrast to the experimental observation that the slide of lipid patches in opposing monolayers conferred the same membrane inter-leaflet viscosity for the friction experienced by the ends of both short and long chain fluorescent lipid analogues (Horner et al. 2013). This finding suggests that the lipid tails in regular biomembranes are too mobile to allow for interdigitation.

Thus, the idea that concrete physical forces at membrane midplane contribute domain to registration does not seem plausible. Rather, the driving force for domain registration is provided by the line tension around the thicker $L_{{\text{o}}}$ domain. Indeed, application of continuum elasticity theory showed that both elastic lipid deformations and rearrangements of lipids at the water exposed rim of the $L_{{\text{o}}}$/$L_{{\text{d}}}$ interface may reduce $\gamma$ (Galimzyanov et al. 2015). These lipid rearrangements include shape change of lipid domains and alignment of domains from opposing leafs. Interestingly, domain registration must be somehow incomplete (Fig. 6), i.e., a small shift between opposing domain edges must persist in order for $\gamma$ to reach its minimum of $\approx$ 0.5 pN (Galimzyanov et al. 2015).

Registration is also promoted by thermal undulations which tend to align stiffer regions from both membrane leaflets (Horner et al. 2009). The simple reason for this is that stiffer lipid domains localise to areas with lower monolayer curvature (Galimzyanov et al. 2017). These areas naturally coincide with opposing monolayers. The required heterogeneity in splay rigidities may originate from intrinsic lipid properties. Alternatively, it may be acquired by adsorption of peripheral molecules (Horner et al. 2009). The energetic contributions from undulations and line tension act hand in hand. Undulations govern the registration of larger domains, since they are proportional to membrane area, whereas line tension dominates the co-localisation of smaller domains, since it is proportional to the domain radius.

### A Dynamic, Water-Mediated Hydrogen-Bonded Network Interconnects Lipids on the Membrane Surface

The interface of the lipid membrane is a complex environment where the lipid headgroups interact with each other, with water, ions, and other molecules that bind to the membrane interface. Membranes that contain anionic lipids are particularly important, because altered distribution of anionic phosphatidylserine lipids can associate with a number of human diseases (Zwaal et al. 2005), and negatively charged lipids are found in bacterial membranes. A key open question here is how cations and positively charged protein regions might bind at the hydrated lipid membrane interface. Atomistic simulations of lipid membranes composed of mixtures of phosphatidylcholine and phosphatidylglycerol lipids (4:1 and 5:1 POPC and POPG) were used to derive a molecular picture of the complex dynamics at the interface of these membranes (Karathanou and Bondar 2018).

**Fig. 7:**
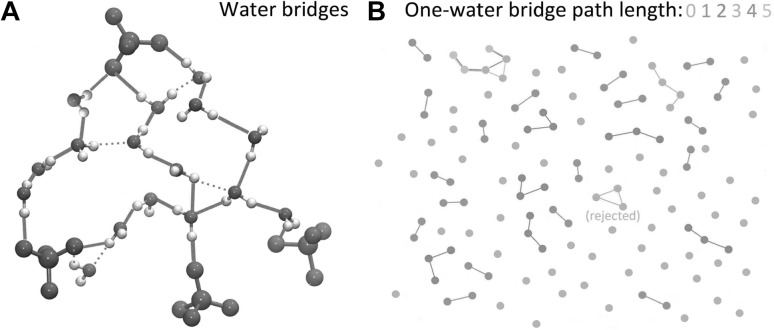
Hydrogen-bonding water bridges between lipid phosphate groups. The data are from a simulation of a membrane composed of 4:1 POPC/POPG lipids (Karathanou and Bondar 2018). **a** Illustration of the algorithm used to identify unique water bridges of the shortest distance between two lipid phosphate groups; green lines indicate the shortest hydrogen bond network found for the configuration used. The maximum number of waters allowed in a hydrogen-bonded chain is 5. **b** Topology analysis of one-water bridges path length interconnecting phosphate groups in the upper membrane leaflet. The visualisation is based on the coordinates of atoms from a simulation snapshot. The dots indicate phosphorus atoms and are coloured as grey, green, red, yellow, magenta, and cyan according to the path length *L* of water-mediated bridges in which they engage. *L* can have the following values: 0 (no bridge), 1, 2, 3, 4, or 5. For simplicity, only linear paths are considered, whereas cyclic paths are excluded from the search. If $L>3$ and cycles are part of a larger path, all possible linear path lengths are computed and the one with the longest length is derived as a result. The image was generated with MATLAB R2017b (The MathWorks, Inc. 2017). Molecular visualisations were prepared with VMD (Humphrey et al. 1996)

To find out how lipid molecules interact with each other, an algorithm to derive the network of connections between lipid headgroups and visualise these interactions was developed. Bridges of shortest distance hydrogen-bonded chains that connect pairs of lipid phosphate groups were searched (Fig. 7a). The analysis can be further extended to characterise the dynamics of larger lipid clusters bridged by hydrogen-bonding water, and the size of lipid clusters of specific topology.

The simulations and analysis revealed that a complex and dynamic network of hydrogen-bonded water bridges characterises the lipid membrane interface with lifetimes on the order of picoseconds. Bridges between lipid phosphate groups, mediated by one hydrogen-bonding water, can form linear clusters, whereby more than two lipid molecules are bridged transiently via water. The linear length *L* of such connections, defined as the number of linearly interconnected phosphate bridges, can be characterised by performing a topology analysis (Figure 7B). During the dynamics, most paths have lengths of 1 or 2, with a significantly smaller probability for the longer linear paths ($L>5$). The two membrane systems studied had similar dynamics of the water hydrogen bond networks (Karathanou and Bondar 2018).

The results of this analysis suggest that the dynamic of the lipid clusters would be altered when a protein or drug molecule binds to the lipid interface. Moreover, the presence of transient lipid clusters could contribute to the propagation of perturbations in membrane structure and dynamics at remote distances from the protein binding site could be affected by the water hydrogen bonding network at the membrane interface. Analyses of water/lipid hydrogen bonding networks and lipid clustering could be extended to account for interactions with cations or for more complex topologies of the transient lipid clusters.

### Transfer of Solutes from Water to Lipid Bilayers

From basic pharmacology to drug design, knowledge of how drugs permeate biological membranes (be it passive diffusion or active transport) and how fast this permeation is, is a crucial aspect when one wishes to develop new drug candidates. For example, when the drugs targets are intracellular, in both eukaryotic and prokaryotic cells, the ability of drugs to cross the membrane highly influences their efficacy. Moreover, membrane proteins are highly targeted by the pharmaceutical industry, due to the wide range of processes in which they are involved. With the progress in technology, computational tools and software, the number of simulations of lipid systems has been increasing. In addition, with the existing force field parameters to describe lipids (parametrised to reproduce experimental data of hydrated lipid bilayers), it is now possible to simulate these systems with increased accuracy.

The partition between water and lipid bilayers of compounds exhibiting antimicrobial properties was studied through an active collaboration between computationally oriented and experimental scientists. These compounds included chelating molecules that may be used to fight infection based on the concept of iron deprivation (Coimbra et al. 2014); and metalloantibiotics that have been developed to bypass known antimicrobial resistance mechanisms (Sousa et al. 2017). Transition metal complexes with zinc, iron, and copper are of particular interest. This is an interesting and somewhat unexplored field, because there are few computational studies addressing the interaction with and transfer of metal complexes to biological membranes. This is probably justified by the inability of classical force fields to describe the dynamics of coordination chemistry in the context of membrane transport mechanisms.

The impact of physico-chemical properties, defined in commonly employed drug-like character predictors (such as the number of rotatable bonds, polar surface area, and size, among others), in the partition and permeation of small compounds is also an interesting venue for research. Veber et al., for instance, noticed that reduced molecular flexibility, defined by the total number of rotatable bonds in a molecule, and a low number of hydrogen bond donors and acceptors (or polar surface area) were important predictors of good oral bioavailability (Veber et al. 2002). Despite the bulk amount of work performed on the impact of molecular/physico-chemical descriptors for membrane permeation, bioavailability, and the drug-like character of compounds, among other, these are still undesirably fallible. Their application could eliminate drugs that might be bioavailable but that are out of the limits of the chemical space of commonly used descriptors. In principle, by employing physics-based methods it is possible to fully disclose the impact of these descriptors to processes such as membrane partition or permeation, drug-receptor binding, and, in the case of MD simulations provide spatially and temporally resolved measurements over these processes (Ribeiro et al. 2017).

**Fig. 8:**
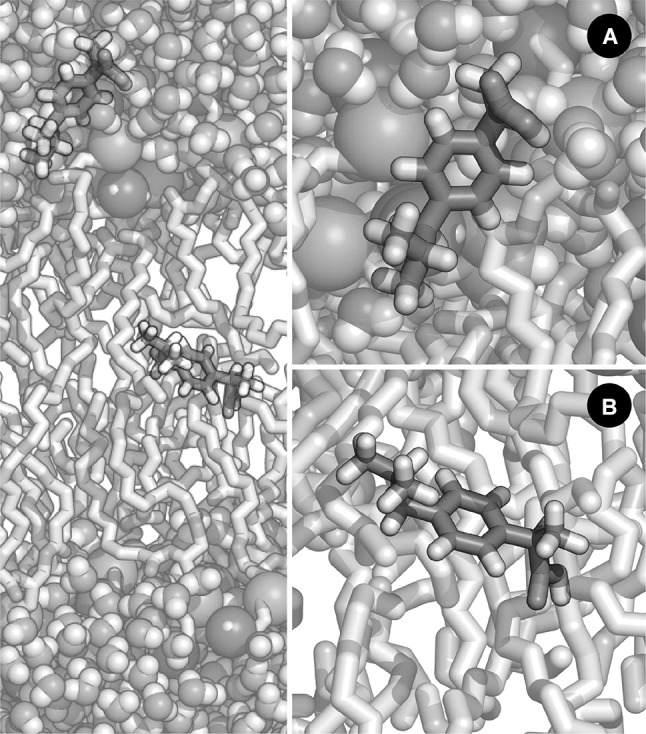
Representation of ibuprofen embedded in a hydrated lipid bilayer system and at two different depths. Two different conformations for the carboxyl acid of ibuprofen have been described (cis and trans), depending on the position relative to the bilayers normal. In this illustration, ibuprofen is depicted while it is **a** in the polar region of the bilayer; and **b** in the centre of the bilayer. The employed set of charges has an impact on the distribution of the dihedral angle of the carboxyl group of ibuprofen, along the membrane normal and thus on the free energy profile for membrane crossing (Coimbra et al. 2017). An accurate portrayal of this distribution is essential for the correct description of its permeation process. In this figure, the membrane and ibuprofen are represented as sticks, while water molecules, phosphorus and nitrogen atoms are represented as spheres. (Phosphorus and nitrogen spheres have been enlarged for illustration purposes.) The following colour scheme was employed: C, white (membrane) or green (ibuprofen); N, blue; P, gold; O, red; and H, white

The transport of molecules across biological membranes is a very complex event that is normally associated with degrees of freedom (DOF) orthogonal to the membrane insertion reaction coordinate. Typically, one needs to resort to enhanced sampling techniques or extended simulation timescales to accurately characterise the energetics associated with these processes. In a recent publication, the impact of the atomic point charges parametrisation to the translocation of ibuprofen was addressed (Coimbra et al. 2017). It was observed that depending on the employed set of charges, the distribution of the dihedral angle of the carboxyl group of ibuprofen (an orthogonal DOF) showed a distinct profile along the membrane normal (Fig. 8). An accurate portrayal of this distribution is essential for the correct description of its permeation process. This work highlighted the necessity of a careful inspection of the atomic charges and that polarisable force fields may represent the best option for this case, as different conformations have a significant effect in atomic charge, and vice versa.

### Mechano-Sensation Through and at Membranes

**Fig. 9:**
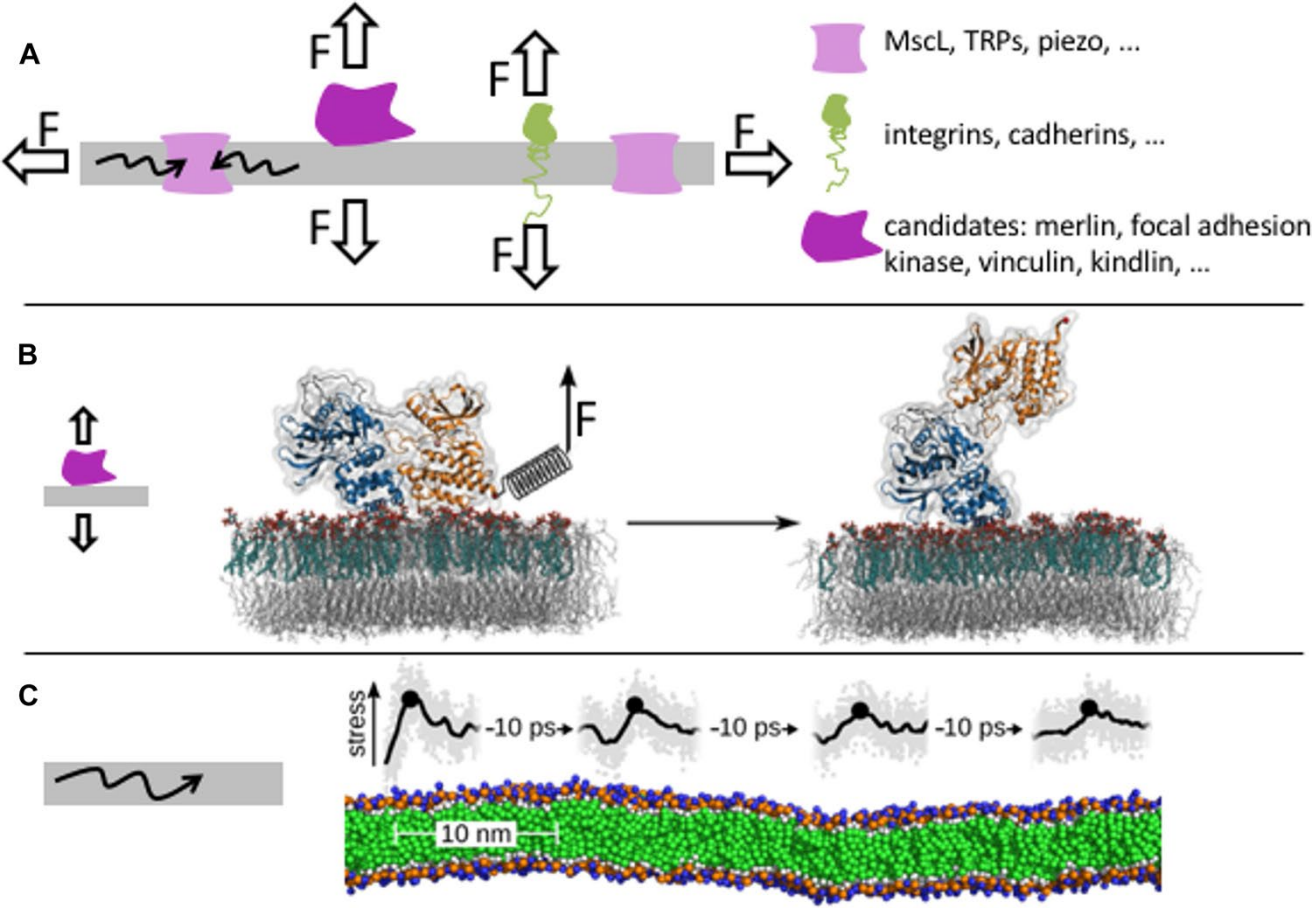
Membranes are directly involved in mechano-sensing. **a** Membranes propagate mechanical force, be it a shearing, stretching, or compressive force, vertical or orthogonal to the membrane, onto protein mechano-sensors such as membrane channels, receptors, or membrane-anchored proteins. **b** Focal adhesion kinase attached to the membrane through PIP2 is activated by mechanical force, as suggested by MD simulations (Zhou et al. 2015). **c** Pressure pulses travel through lipid bilayers over many tens of nanometers at the speed of sound (Aponte-Santamaría et al. 2017)

Membranes are an integral part of cellular mechano-transduction pathways. Critical receptors that connect the cellular cytoskeleton to the extracellular matrix or the neighbouring cell, be they integrins or cadherins, as well as mechano-sensitive ion channels (Ranade et al. 2015) are spanning the membrane and thus are directly subjected to membrane mechanical forces. Mechanical stress can act vertically or in parallel to the membrane plane and tightly regulate the function of these and other integral membrane proteins (Fig. 9a). It is less obvious that the membrane also plays an essential role in propagating force through membrane-anchored proteins. Interestingly, many integral focal adhesion components are membrane bound. These include focal adhesion kinase (FAK), Src kinase, kindlin, vinculin, paxillin, and many others. An important question is how force propagates through membranes to eventually arrive at integral and to anchored mechano-sensitive proteins. If the protein is only anchored, either through covalently attached lipid anchor or through a non-covalent interaction with specific lipids, e.g., phospho-inositol (4,5)-bisphosphate (PIP2), to the lipid, the question arises if force leads to a dissociation of the protein from the membrane or to a functionally relevant conformational transition of the protein.

FAK is an important component of the focal adhesion complex, a cell-extracellular matrix junction. In equilibrium, FAK is not catalytically active, since the kinase domain is inhibited by the FERM domain (4.1 protein, ezrin, radixin, and moesin), which is the N-terminal domain of the same protein. This autoinhibition can be released through at least two mechanisms. First, binding to PIP2 and phosphorylation trigger activation and clustering of FAK, as has been shown experimentally (Goñi et al. 2014). The second mechanism similarly requires binding to a sufficient concentration of PIP2, but involves instead mechanical force, as have been suggested by simulations (Zhou et al. 2015). By combining MD simulations and biochemical network analysis, the effect of force propagation onto lipid-bound FAK structure and function has been investigated. It was demonstrated that tensile forces unlock the central phosphorylation site of FAK, thereby triggering its activation, while it remains tethered to a PIP2 cluster at the membrane (Fig. 9b). On the contrary, at insufficient concentrations of PIP2, FAK simply detaches from the membrane when under force. FAK thus emerges as a potential mechano-sensor and PIP2-enriched membranes as key adhesion anchor points that enable FAK to broadcast force signals through to the nucleus.

The PIP2-mediated clustering of FAK and subsequent activation is a remarkable finding, pointing to the possible role of FAK not only an enzyme but also as a recruitment and scaffolding factor for focal adhesion assembly. Bearing in mind that focal adhesion assembly is tension dependent, an intriguing question to be further clarified is how the two pathways of activation (mechano-sensing and clustering) interact during the maturation phase of focal adhesions. Ultimately, further understanding the behaviour of membrane-bound adhesion proteins, including vinculin, kindlin and others, under the conditions of clustering and mechanical stress could help us uncover the full order of focal adhesion maturation.

Mechanical stress also propagates laterally through the membrane to thereby trigger the activation of mechano-sensitive channels (Ranade et al. 2015). MD simulations, this time at different levels of resolution varying from all-atom to CG, helped to clarify how fast and how far mechanical stress propagate laterally through model mono-lipid bilayers (Aponte-Santamaría et al. 2017). The calculations revealed that nanometer-wide localised pulses of mechanical stress efficiently propagate at speeds of nm / ps for up to several tens of nanometers before attenuation (Fig. 9c). The results suggested that these pulses could play a key role coupling mechano-sensitive elements in crowded membranes, such as those present at focal adhesion sites, in which the membrane spacing reduces to nanometers.

Future experimental and simulation studies will hopefully help to unravel in further detail the active role of membranes in mechano-sensing. Intriguingly, membrane mechanical properties are tightly regulated through differential lipid compositions along the plasma membrane of the same cell (Atilla-Gokcumen et al. 2014). Exciting new insights on how biological membranes spatially and temporarily distribute mechanical forces among the attached proteins inside and outside of the cell are to be expected in the near future.

## Membrane Proteins and Their Regulation

Membrane proteins have to operate inside the membrane, while they often also have extra- and intracellular parts. Many membrane proteins have tremendous significance in biology. Yet, following on their dynamics can be elusive: physical differences between the lipid and aqueous environments can make it difficult to gain experimental insights directly by biophysical methods. It is especially interesting—and challenging—to learn how membrane proteins are regulated by gating, how they combine with other proteins, and how they degrade. Importantly, it has been shown that cellular lipids may to some extent be involved in many severe diseases such as cancer, psychiatric dysfunction, and type 2 diabetes mellitus (T2DM). The systems discussed here exemplify the advantages of studying membrane protein regulation using simulations or bioinformatics.

### Regulation of Rhomboid Intramembrane Proteolysis and Substrate Gating

**Fig. 10:**
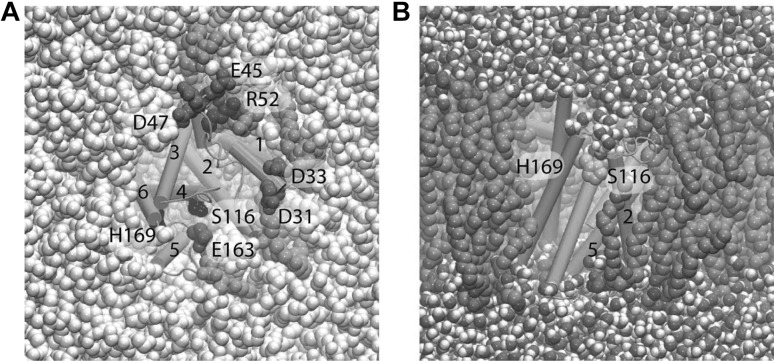
*H. influenzae* GlpG in a hydrated lipid membrane environment. **a** View from the periplasmic side showing selected protein groups and lipid molecules. The catalytic S116 and H169 are depicted as black van der Waals spheres; selected Asp/Glu and Arg/Lys sidechains are coloured red and blue, respectively. Lipid molecules whose headgroups are within hydrogen bond distance of the carboxylate, lysine and arginine sidechains shown are coloured green, other lipid molecules are in grey. Water molecules at the cytoplasmic side of the membrane are coloured pink. **b** Side view of *H. influenzae* GlpG in POPE, showing that lipid alkyl chains can intercalate in the interhelical space. Molecular graphics were prepared with VMD (Humphrey et al. 1996)

Rhomboids are a family of evolutionary conserved intramembrane serine proteases (Düsterhöft et al. 2017). They function to cleave transmembrane proteins playing a role in cell signalling events. For example, in Drosophila, the cleavage of tethered epithelial growth factor (EGF) from the plasma membrane of follicle cells influences cell differentiation during development. Another example is the cleavage of the PINK1 kinase in mitochondria, which prevents signalling for cell death. Rhomboid proteases have a core catalytic structure of six-transmembrane segments that form an $\alpha$-helical bundle (Wang et al. 2006). The catalytic serine becomes active by a general base histidine. Together they form a catalytic dyad, which is buried approximately 10 Å below the surface of the bilayer (Fig. 10). The mode of cleavage of transmembrane substrates, and in particular how they gain access to a buried active site, has always been one of the central questions in the rhomboid field (Brooks et al. 2011). While some studies have shown that the substrate cleavage site is accessible from the aqueous channel on the extracellular face of the enzyme (Strisovsky et al. 2009), there is structural and kinetic evidence that transmembrane helices in the enzyme must be dynamic to allow access laterally for transmembrane substrates (Arutyunova et al. 2014). Interestingly, transmembrane helices 5 and 2 harbour strong $\pi$-stacking interactions, via Trp-Phe-Phe residues, that block accessibility to the active site. Preliminary MD simulations of *H. influenzae* GlpG embedded in a hydrated POPE lipid bilayer suggest that protein/lipid interactions (Fig. 10a) shape the conformational dynamics of GlpG in the membrane. Reducing the $\pi$-stacking of helices 2 and 5 via mutation is associated with enhanced conformational dynamics at the gate region. It is logical to assume that once these two helices are separated, the enzyme may become more active if a substrate enters laterally. Preliminary mutagenesis studies confirmed this hypothesis and demonstrated that the cleavage rate for transmembrane substrate is indeed enhanced when these helices are separated, while the cleavage rate for soluble substrates remained unchanged. Furthermore, MD simulations demonstrate that during gate opening, the POPE lipid can fit into the cleft occupying a previous transmembrane helix. The data hint towards different regulatory modes for substrate gating depending on the substrate and the environment.

### Membrane Lipid–GLUT1 Transporter Interactions Explain the High Temperature Sensitivity of Glucose Transport

d-Glucose is known to be specifically transported via transmembrane proteins across biological lipid membranes. The main functional attributes of passive facilitated glucose transporters, i.e., ligand specificity, saturability of transport rates, and asymmetric transport, have been ascribed to the intrinsic properties of the transporter, independent of its membrane support. A lesser explored aspect of passive glucose transport is its particular sensitivity to temperature. The maximal rates of glucose net exit flux from and influx into human erythrocytes decrease abruptly below $\approx$ 24 °C, corresponding approximately with the onset of gel phase change of the membrane lipids. Similar in vitro correlations exist with glucose transporters reconstituted into large unilamellar lipid membrane vesicles made from DPPC bilayers, among other lipids (Tefft et al. 1986). These in vitro model systems demonstrated that net glucose transport rates correlated with the gel-fluid phase transition temperature of the membrane lipids. With DPPC bilayers, net transport glucose transport fell to virtually zero in the gel phase and rose abruptly on raising the temperature to affect fluid phase transition of the bilayer. Thus, it is evident that forces exerted by the membrane on the transporter play a role in glucose transport.

The single site alternating access model for passive glucose transport describes how the glucose ligand is transported by binding first to a centrally located high affinity site accessible to the external solution. This model has received powerful support from structural studies. Isoform crystal structures consisting of open out, occluded, and open isoforms in both apo and holo forms exist for several of members of the major facilitator superfamily (MFS) (Deng et al. 2014). Another view is that glucose binds and dissociates from multiple sites within the central channel traversing the transporter in the cleft between the N- and C-terminal halves. Small local conformational changes occurring in the nanosecond time domain permit opening and closure of intramolecular cavities containing these binding sites, thereby permitting a staged diffusion of glucose to transit the entire channel. Demonstration of docking sites in the exofacial and endofacial vestibules, in addition to the central high affinity site, has been observed by static and dynamic docking and mutation studies (Cunningham et al. 2006). This accounts for the high temperature sensitivity of glucose transport lend support to this model.

The multisite mechanism for passive glucose transport, unlike the alternating access model, does not necessarily require large conformational changes within the transporter. It does, however, imply that both the susceptibility of the intramolecular cavities and tunnels to lateral pressure from the lipid membrane, and the intrinsic flexibility of the ligand, may have significant roles in transport. This might explain why transport correlates with lipid fluid-gel phase change (Tefft et al. 1986). It also may account for the finding that the *β*-d-glucose anomer is transported more rapidly than the $\alpha$-anomer via GLUT1 (Duan et al. 1992), the reason being that *β*-d-glucose has a lower enthalpy barrier than *α*-d-glucose to reach the conformation where the radius is minimal (puckered $^{1} {\text{S}}_{3}$ skew boat) (Mayes et al. 2014).

A demonstration that the bottlenecks within the transport channel should transiently open sufficiently to permit glucose passage is required to test the validity of the staged diffusion model of glucose transport. MD simulations of GLUT1 embedded in bilayers of dipalmitoylphosphatidylcholine (DPPC) at temperatures below and above its gel-fluid phase transition temperature were performed to observe the effects on the volume changes in the intramolecular tunnels and cavities. When the protein was embedded in the gel phase membrane, the dimensions of the intramolecular pathways were reduced (Iglesias-Fernandez et al. 2017). The maximum value of the bottleneck radius found in the inward-facing branch of the pathway, in either the gel or fluid phase, permits the passage of glucose molecules with a minimal radius of 1.9 Å. In the gel phase membrane, the outer gate was found to have a maximal radius of 1.54 Å. However, crucially, in the fluid phase, this bottleneck widens occasionally so that glucose can gain access to the inner parts of the transporter from the external solution.

The gel to fluid phase change reduces the membrane bilayer average thickness from 43 to 41.5 Å. Membrane thickness correlates negatively with the channel bottleneck radius (*r* = −0.54, *p* < 0.001). Additionally, the gel to fluid phase change increased the membrane surface area from 69 to 72 Å$^2$/lipid, and the membrane area correlates positively with bottleneck radius (*r* = 0.66, *p* < 0.001).

It is apparent that membrane fluid to gel transformation compresses of the transporter, as maintaining the transporter temperature at 50 °C, while cooling the membrane lipids to 35 °C had a similar effect to cooling both membrane and transporter to 35 °C (Iglesias-Fernandez et al. 2017).

The effects of temperature on the lipid-order parameters are not uniformly distributed within the membrane. The largest changes occur in the carbon chains midway between C3 and C14 ($\approx \varDelta 0.175$) at a distance > 3 Å from the protein, whereas, at distances < 3 Å from the protein, lesser changes in the membrane lipid order occur ($\approx \varDelta 0.11$). These findings, only accessible by MD, indicate that in the gel phase, GLUT1 exerts a force that reduces the neighbouring lipid order parameters. Thus, it is evident that the transporter also exerts temperature-dependent lateral forces that alter the order parameters of the membrane lipid acyl chains.

### Membrane Dynamics Can Modulate the Association of Transmembrane Receptors

The ErbB growth factor receptor family forms a critical hub in controlling cell growth, making it an important target in several cancers. The dimerisation of ErbB receptors is an essential step in their function and was thought to be protein or ligand driven. Increasingly, effects that depend on lipids have been reported on the association, and consequently function of these receptors. Understanding the interactions of these receptors with membranes is thus an emerging area and assumes greater significance in light of altered membrane composition in several cancers.

Lipid effects reported for membrane proteins in general, and ErbB receptors in particular, can be classified into direct and indirect effects, based on whether the membrane lipids specifically interact with the receptor, or exert their effect by modulating the environment (Pawar and Sengupta 2018). Due to the inherent resolutions of current experimental methods, analysing the molecular determinants of both specific and non-specific effects has remained challenging, and computational methods are helping to bridge the gap. An important indirect effect, namely the “lipophobic effect”, has been reported in several membrane proteins (Duneau et al. 2016; Sengupta and Marrink 2010). The lipophobic effect refers to the energy differences in a lipid bilayer due to the embedded proteins and results in increased association of membrane proteins.

**Fig. 11:**
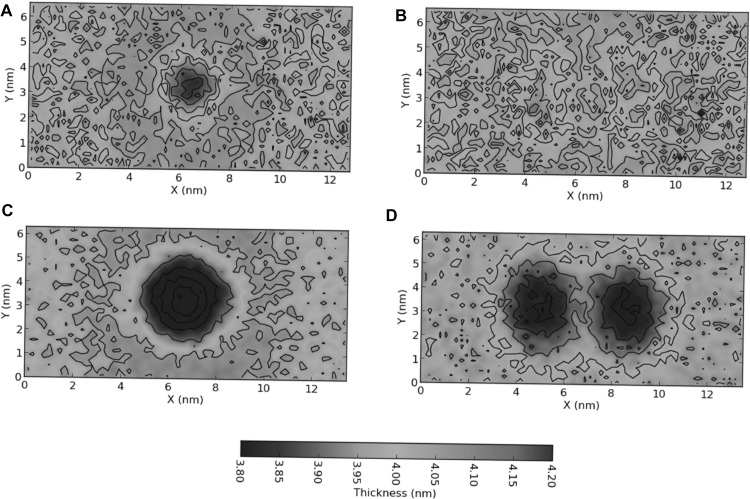
Modulation of membrane thickness around a transmembrane receptor. Two-dimensional membrane thickness profiles around the ErbB2 growth factor receptor transmembrane domain, calculated as described in (Prasanna et al. 2013). The membrane thickness corresponds to the transmembrane dimer (left panel) and the two spatially separated monomers (right column) of **a**, **b** wild-type ErbB2 and **c**, **d** the V659E/V664E associative mutant. The green/yellow stretches correspond to the bulk membrane thickness, and the blue stretches correspond to membrane perturbations resulting in decreased membrane thickness

The lipophobic effect in the ErbB2 receptor was highlighted when comparing the free energy of dimerisation of a series of putative oncogenic mutants (Prasanna et al. 2013). CG simulations and umbrella sampling calculations reported differences in the dimersation free energy. The calculated values of free energy calculated were in line with their experiment values (Beevers and Kukol 2006; Beevers et al. 2010, 2012; He and Hristova 2008). Despite differences in the free energy profiles, the protein–protein interaction energetics of these mutants were similar. Interestingly, membrane perturbations arising from differences in membrane thickness were observed around the transmembrane domain of the receptor (see Fig. 11). These local perturbations were larger in the vicinity of the associative mutants (for instance the V659E/V664E double mutant) than near the wild-type receptor, but were minimised when the receptors dimerised. Consequently, lipid perturbations caused by the transmembrane domains were identified as the main driving force of dimerisation (Prasanna et al. 2013).

A direct estimate for the lipophobic effect is difficult, but initial calculations based on insertion of model peptides have identified significant contributions (Dubey et al. 2017). The values were calculated from the cost of cavity formation using coarse-grain simulations and ranged between 50 and 150 kJ/mol for a single residue. Similarly, lipid perturbations have been reported for larger membrane proteins that were related to the lipophobic effect (Katira et al. 2016; Duneau et al. 2016), although a direct estimate is still lacking. The values have been estimated for single component bilayers and are expected to be lower in mixed component membranes. The experimentally observed lipid composition and fluidity effects in transmembrane protein association could arise due to the lipophobic effect, but further work is required to obtain a direct estimate. Understanding the roles of the membrane in receptor association will allow us to delineate general design principles underlying receptor organisation.

### The Membrane Physical State Affects Which Peptides are Connected to it

The activity of membrane proteins is influenced and controlled by the chemical and physical properties of the lipid mixture (Török et al. 2014; Casas et al. 2017). The proportions and types of membrane lipids define different types of membrane microdomains that regulate: (i) the activity of the proteins; (ii) their localisation; and (iii) protein–protein interactions ensuring, therefore, signal propagation. Consequently, the activities of many membrane-associated proteins and transporters also change dramatically. It is conceivable that molecules capable of interacting with membrane lipids may induce modifications in membrane composition, protein function or gene expression and reversion of the pathological state.

It is well known that when temperature changes, cells produce adaptive responses. Recently, a new hypothesis suggests the role of the membrane as a sensor (Török et al. 2014), in which the remodelling of microdomains and the physical properties of the membrane are involved in the heat shock response (HSR) without protein denaturation. Changes in membrane lipid composition and altered heat shock protein levels are often found in diseases such as diabetes, cancer or neurodegenerative illnesses. The function of transmembrane (TM) proteins depends on the lipid environment. Composition analysis of TM segments of membrane proteins has shown that they are dominated by hydrophobic amino acids such as Ile and Val, but the exact composition reflects, among others, the type of aggregation and the lipid environment. All these aspects raise several important questions: how is a TM protein selected for a particular lipid environment? What happens to the peptide–membrane interaction when the system is physically or chemically altered, for example by changing the temperature, pressure, lipid composition or upon the effect of a pathogen?

The interplay between transmembrane regions and the lipid environment becomes even more evident if we consider thermophiles. Thermophiles are bacteria or archaea that live at temperatures above 60 °C. In some cases, they can survive at temperatures higher than 100 °C and high pressures. Those organisms, regardless of their evolution, learn to cope with high temperatures using special lipids that have high transition temperatures; such lipids cannot be found in cells living at lower temperatures. How do thermophiles adapt the amino acid composition of their TM portions to deal with exotic lipids? The understanding of the interplay between the peptide sequence and the membrane physical state (MPS) is facilitated by the study of the membrane composition of thermophiles. Organisms living at high temperatures require more rigid membranes, and the TM peptide sequences must be different to ensure the best interaction. Therefore, the peptide function can be understood in terms of both the sequence and the MPS. The MPS is in itself a function of temperature and lipid composition.

If it is assumed that specific peptides are well adapted to a particular MPS to show a specific function, then it should be possible to use sequence analysis to infer on the MPS that, perhaps more than the lipid composition, is the driving force of peptide–membrane interactions. The MPS can also control protein localisation and peptide self-assembly. For example, the dimeric $\text{G}\beta \gamma$ protein complex associates preferentially with membrane or membrane regions that adopt the $L_{{\text{d}}}$ phase through the isoprenylated tails of the $\text {G}\gamma$ subunit. $\text{G}\alpha$ subunits, on the other hand, bind better to membranes or membrane regions which adopt the $L_{{\text{o}}}$. (Casas et al. 2017; Piotto et al. 2014). Let us consider the transmembrane portion of a protein. It is reasonable to assume that a particular sequence has been selected by the evolution to offer the best interactions with other parts of the protein, or with the membrane, in a given condition. In fact, the integration into a bilayer is simply a matter of hydrophobicity and can be achieved with few amino acids only. The helicity of a peptide can be obtained with the regular distribution of residues, whereas the ability to self-assemble into a pore, for example, requires a sophisticated adaptation to that particular lipid environment. Hence, an approach which exploits novel metrics to calculate similarity among sequences has been proposed, where transmembrane peptides are used as a sort of bioinformatics sensors of the MPS.

**Fig. 12:**
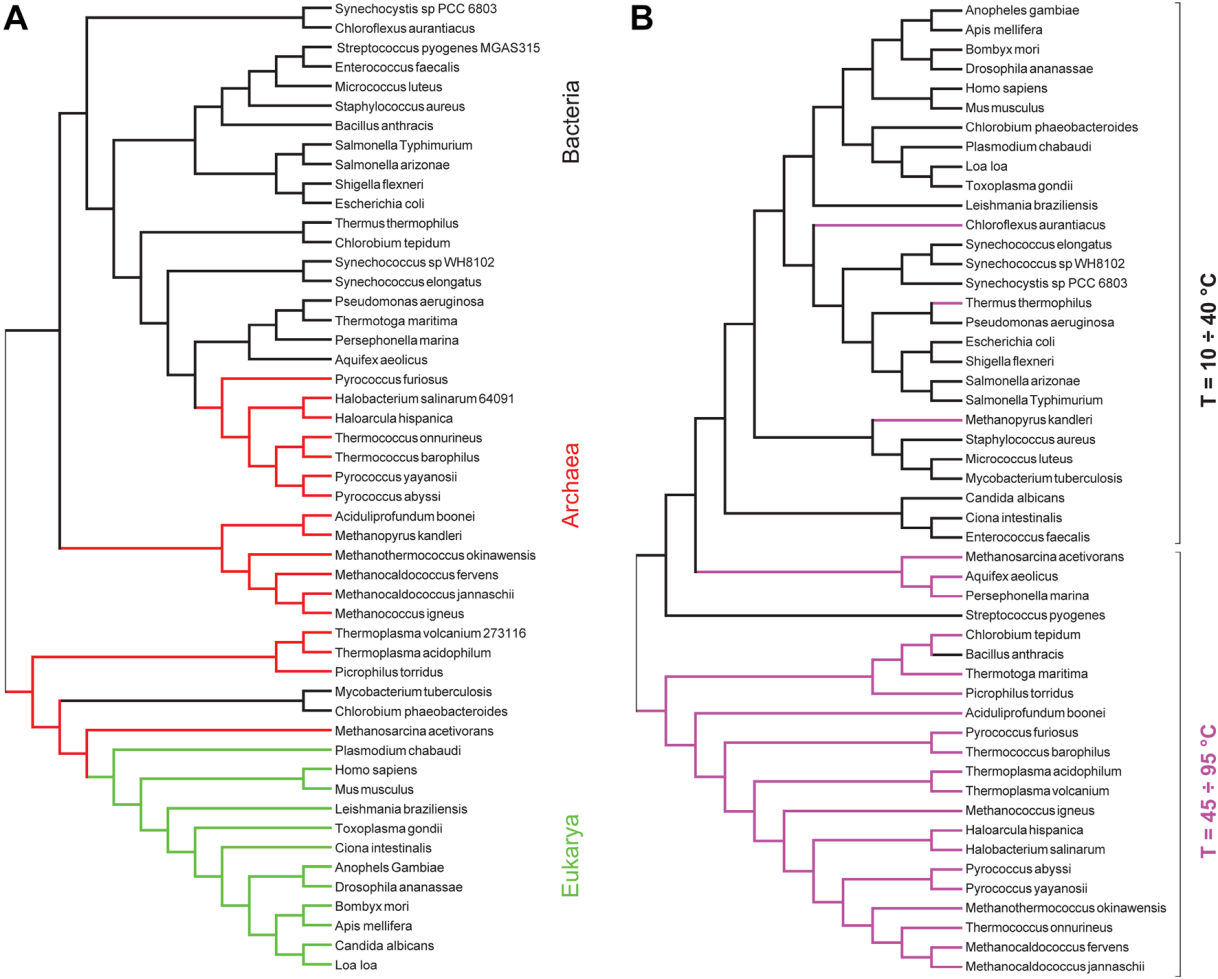
Evolutionary relationships of full and transmembrane-only proteomes. **a** Evolutionary relationships of proteomes. Eukarya are shown in green, bacteria in black and archaea in red. **b** Evolutionary relationships of TMP. Thermophiles are shown in purple. The data are from Piotto et al. 2018

To apply this approach, 51 proteomes have been processed with Phobius (see Fig. 12), and the collection of all predicted transmembrane sequences of a given proteome was called transmembrane proteome (TMP). Consequently, there is a TMP for any proteome. An alignment-free approach was chosen to analyse the peptide sequences. The proposed metrics have been implemented in the software ProtComp. ProtComp consists of python scripts to parse the input files and prepare them for the analysis. The preprocess script deals mainly with the removal of all unneeded data, like FASTA headers. A second script permits the extraction of substrings of length k (k-mer).

During the calculation of the similarity between two sequences, the weight of the feature, expressed as the number of occurrences, determines the similarity and not the position of the conservative features. The evolutionary history was inferred using the minimum evolution (ME) method. The ME tree was searched using the close-neighbour-interchange (CNI) algorithm (Nei and Kumar 2000). The neighbour-joining algorithm was used to generate the initial tree. Evolutionary analyses were conducted in MEGA7 (Kumar et al. 2016). Of note, alignment-free methods on whole proteomes do not rely on one of few proteins to derive the phylogeny, and the results can differ from those conducted on one gene only. This aspect is particularly critical for the determination of the position of thermophiles in bacterial phylogeny.

A phylogenetic tree obtained by the ProtComp analysis is shown in Fig. 12a. [More details are available in Piotto et al. (2018).] The calculation of the distance matrix among 51 full proteomes is extremely fast and lasts few seconds on i3 processor. It permitted to cluster clearly the organisms in different phyla. Following the hypothesis that transmembrane portion of proteins might be well adapted at the particular chemical-physical environment in which they work, the same analysis was conducted with transmembrane proteomes. Figure 12b represents the ME tree of TMP. In contrast to Fig. 12a, the organisms did not cluster according the phylogenetic relationships. The TMP is organised in a sort of temperature ramp with organisms living at ambient temperatures separated from those living in extreme conditions. The need to adapt to high temperatures poses a dramatic challenge to the cells that cannot use ordinary lipids for their membranes. In fact, the living temperature cannot be more than few degrees hotter than the transition temperature of the lipid mixture. It is important to recall the fact that transmembrane portions of proteins contain a high percentage of hydrophobic residues (necessary to accommodate in membranes) and few or no charged amino acids. Consequently, the alphabet of TMP is limited if compared with that of proteomes. The metrics reported here could successfully discriminate among TMP regardless of the limited compositional variability.

In conclusion, the MPS depends on the local lipid composition that is, more often than not, not accessible experimentally. Moreover, MPS changes with temperature and with the cell phase. An alignment-free method had been used to calculate the similarity of strings. This approach was applied to proteomes and correctly reproduced known phylogenetic trees. At the same time, when this approach was applied to the transmembrane proteomes, it enabled the observation of evolutionary relationships among organisms sharing the same environment. This is, so far, the first quantitative observation of coevolution of transmembrane portions. The transmembrane sequences reflect the MPS, and they can be used as an indirect measure of the lipid composition. Since this approach proved its efficacy in determining indirectly the differences among membranes of different organisms, it can also be used for inferring the possible interaction of a given peptide with a target membrane. Taken altogether, these data and this approach reveal the fundamental role of lipids in organism evolution. Moreover, the method offers unprecedented possibility to design better (more selective) antimicrobial peptides.

Protocomp is available at:

www.yadamp.unisa.it/protcomp

### Amylin Binding and Aggregation—The Role of Negatively Charged Lipid Headgroups

The cellular membrane is a complex mixture of lipids with embedded peptides and proteins. It has become evident that the lipid composition may affect the function of the embedded peptides and proteins, and it has been shown that cellular lipids may to some extent be involved in many severe diseases such as cancer, psychiatric dysfunction, and type 2 diabetes mellitus (T2DM). Experimentally, it is an extremely demanding task to study how lipids influence protein and peptide function; hence, simulations have emerged as a technique that may provide pivotal insight to this intriguing question.

T2DM is characterised as a loss of insulin response leading to a decreased uptake of glucose. The beta cells of pancreatic islets are the cells that produce insulin and islet amyloid polypeptide (IAPP, or amylin), two hormones that regulate the glucose metabolism. In patients with T2DM, amyloid fibrils of IAPP are formed at the extracellular surface of beta cells. As T2DM progresses, the amount of beta cells is reduced, leading to a further decrease in regulation of the glucose metabolism. From cell studies, it has been observed that high concentrations of IAPP are cytotoxic, and in vitro IAPP can cause dye-leakage from lipid vesicles. The mechanism of cytotoxicity is unknown, but studies indicate that an oligomer form of IAPP, rather than mature amyloid fibrils, is cytotoxic by damaging the membrane, and that negatively charged lipids accelerate the formation of the damaging species.

IAPP is an intrinsically disordered peptide, meaning that it alternates between multiple conformations in solution. Upon membrane binding, IAPP is stabilised in an $\alpha$-helical conformation prior to forming amyloid fibrils, rich in $\beta$-sheets. At physiological pH, IAPP is cationic and attracted to anionic lipids. The presence of vesicles containing anionic lipids drastically accelerate the formation of amyloid fibrils by IAPP.

**Fig. 13:**
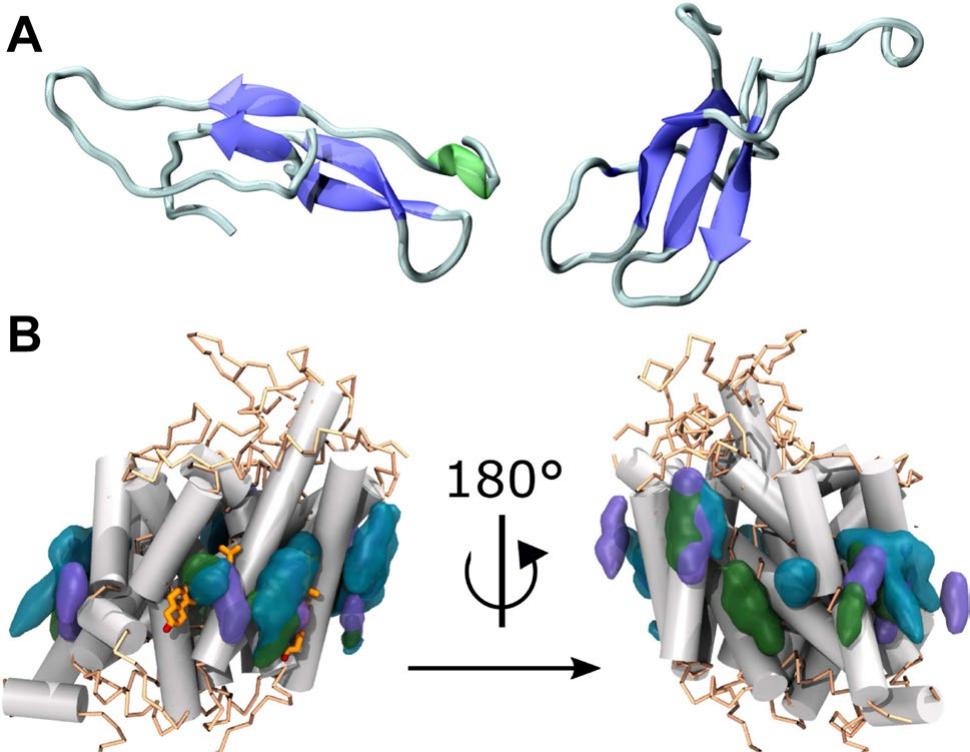
Two examples of systems where lipids affect the dynamics of peptides. **a** Two IAPP dimers formed on a lipid membrane (not shown) in atomistic MD simulation. The peptides are coloured according to secondary structures with coils or turns in grey, $\beta$-strands in blue, and helices in green. **b** Cholesterol occupancy maps. Overlay of cholesterol occupancies as extracted from the three MAT CG simulations: the human dopamine transporter (violet), the human serotonin transporter (cyan) and the human norepinephrine transporter (green). The two co-crystallised cholesterol molecules revealed in dDAT crystal structures are highlighted in orange licorice

MD simulations were carried out to investigate the binding and self-assembly of IAPP on lipid bilayers with zwitterionic and anionic lipids in atomistic detail (Skeby et al. 2016) using the highly mobile membrane mimetic (HMMM) model (Ohkubo et al. 2012). Starting from a membrane-bound structure, systems were constructed with four IAPPs to investigate the interactions between the peptides and the self-assembly process (Christensen et al. 2017). A transition from membrane bound $\alpha$-helical IAPP monomers to $\beta$-sheet containing oligomers was observed in the simulations. When the IAPPs started to interact, the helical structure was destabilised and replaced by interactions with other peptides. The initial contacts were in the C-terminus, with the aromatic F23 residue and later in the highly amyloidogenic 22-NFGAIL-27 segment. The assembly of peptides led to the formation of $\beta$-strand conformation in two regions of the peptide, 14-NFLVH-18 and 25-AILSST-30, corresponding to the regions expected to be in the $\beta$-sheet of mature IAPP fibrils. Examples of IAPP dimers formed on the membrane during the simulations are shown in Fig. 13a.

### Cholesterol and the Human Dopamine Transporter

The human dopamine transporter (hDAT) is essential for regulating dopaminergic neurotransmission by transporting dopamine from the synaptic cleft back into the presynaptic neuron. Dysregulation of hDAT is involved in several debilitating diseases such as Parkinson’s disease, attention deficit hyperactive disease (ADHD) and Tourette’s syndrome. hDAT is also the target of many illicit drugs and is suggested to be involved in the development of addiction. It is therefore essential to gain further understanding in how this transporter is regulated.

It has been shown experimentally that DAT, and other members of the monoamine transporter (MAT) family, are regulated by cholesterol presumably in a direct manner. MAT operate by the alternating access mechanism, meaning that they are only accessible to one side of the membrane at the time. It has been suggested that cholesterol stabilises the conformation in which the transporters are accessible to the extracellular environment (outward-facing conformation). A range of *Drosophilia melanogaster* DAT (dDAT) crystal structures were solved in an outward-facing conformation (Wang et al. 2015). These structures contain one or two conserved co-crystallised cholesterol molecules bound at the protein surface. In combination with the experimental studies showing that cholesterol acts in regulating hDAT activity and results indicating that this occurs in a direct manner, this suggests that the two co-crystallised cholesterol molecules may have a functional role, which could be conserved across the whole MAT family.

CG MD simulations of each MAT protein embedded in a mixed POPC and cholesterol membrane in a 4:1 ratio were used to map preferential cholesterol binding to delineate this hypothesis (Zeppelin et al. 2018). The simulations revealed several cholesterol–protein binding and unbinding events, and it was suggested from the simulations that the sites present in the dDAT crystal structures are conserved across the entire human MAT family (Fig. 13b). In addition, three subsequent conserved cholesterol high occupancy sites were detected. To further study how the conserved co-crystallised cholesterol molecules may influence hDAT dynamics, all-atom simulations of hDAT were carried out. hDAT was simulated in an outward-facing conformation both with and without the two co-crystallised cholesterol molecules bound. From several repeat simulations of the two systems, it was observed that when cholesterol was present at the interface between helices 1, 5 and 7, no indications of conformational transition occurred. On the contrary, without cholesterol transmembrane helix 5 started to unwind and kinked out towards the surrounding lipid milieu, which allowed the formation of a water channel to reach the location of the coordinating Na^+^ ion bound to the Na2 site. These collective events were taken as indicators of early conformational hDAT transition towards a more inward-facing conformation. This study thereby highlights the importance of considering the surrounding membrane environment as a potential regulatory mediator of membrane protein function.

## Conclusions

Computer simulations have been used since more than 40 years ago in the study of lipid membranes. As exemplified by the collection of topics discussed here, the field has in the recent years moved to dealing with more complex mixtures on the one hand, and towards gaining a better understanding of how lipids interact with their environment (proteins, salt ions, water, and drug molecules) on the other. In addition, a purely theoretical study of membrane domains ("Registration of Lipid Domains from the Two Membrane Leaflets") and a study where peptide–membrane interactions were inferred from bioinformatics ("The Membrane Physical State Affects Which Peptides are Connected to it") were also discussed.

Many of the studies that were surveyed in the CECAM meeting and in this paper involved CG forcefields. Together with state-of-the-art computer resources, such models enable the study of complex biological protein-membrane associations ("Modelling the Complexity of Thylakoid Membranes"). As discussed in the meeting, with better computers that are becoming available, larger systems containing dozens of lipids and hundreds of proteins are expected to be within reach. As many of the contributions here discuss, atomistic simulations are often used as reference for the CG simulations. A polarisable force field was used only in one of the studies presented here ("Coarse-Grain Approaches to Study Block-Copolymer Nanoparticles for Drug Delivery Systems").

Beyond the traditional membrane or membrane proteins studies that were reported, several works concentrated on less well-studied systems. These included lipid droplets ("Towards a Molecular View of Lipid Droplet Biology") and nanoparticles ("Coarse-Grain Approaches to Study Block-Copolymer Nanoparticles for Drug Delivery Systems"). Such systems are still more challenging to build using existing tools. A similar topic was addressed by developing a tool that makes it easier to study membranes of various curvatures ("The Effects of Complex Curvature Environments in Heterogeneous Lipid Mixtures").

MD simulations were used to explain experimental findings reported for single proteins ("Mechano-Sensation Through and at Membranes", "Regulation of Rhomboid Intramembrane Proteolysis and Substrate Gating", "Membrane Lipid–GLUT1 Transporter Interactions Explain the High Temperature Sensitivity of Glucose Transport", "Membrane Dynamics Can Modulate the Association of Transmembrane Receptors", and "Cholesterol and the Human Dopamine Transporter"), dimers ("Membrane Dynamics Can Modulate the Association of Transmembrane Receptors"), or small oligomers ("Amylin Binding and Aggregation—the Role of Negatively Charged Lipid Headgroups") with membranes, where experimental evidence alone is not sufficient to provide a complete understanding because some details are not accessible to the experiment. Bilayer membrane systems continue to attract attention, including studies of physical processes such as domain formation ("Domain Formation in Lipid Membranes, Studied by Lattice Models"), domain registration ("Registration of Lipid Domains from the Two Membrane Leaflets"), bilayer curving ("The Effects of Complex Curvature Environments in Heterogeneous Lipid Mixtures"), and interactions with water and solutes ("A Dynamic, Water-Mediated Hydrogen-Bonded Network Interconnects Lipids on the Membrane Surface" and "Transfer of Solutes from Water to Lipid Bilayers"). Overall, computer simulations have become an integral part of biological membrane studies.
